# The Minimum Clinically Important Difference of the Patient-rated Wrist Evaluation Score for Patients With Distal Radius Fractures

**DOI:** 10.1007/s11999-015-4376-9

**Published:** 2015-06-04

**Authors:** Monique M. J. Walenkamp, Robert-Jan de Muinck Keizer, J. Carel Goslings, Lara M. Vos, Melvin P. Rosenwasser, Niels W. L. Schep

**Affiliations:** Academic Medical Centre, University of Amsterdam, Meibergdreef 9, 1105 AZ Amsterdam, The Netherlands; Department of Orthopedic Surgery, Columbia University Medical, New York, NY USA

## Abstract

**Background:**

The Patient-rated Wrist Evaluation (PRWE) is a commonly used instrument in upper extremity surgery and in research. However, to recognize a treatment effect expressed as a change in PRWE, it is important to be aware of the minimum clinically important difference (MCID) and the minimum detectable change (MDC). The MCID of an outcome tool like the PRWE is defined as the smallest change in a score that is likely to be appreciated by a patient as an important change, while the MDC is defined as the smallest amount of change that can be detected by an outcome measure. A numerical change in score that is less than the MCID, even when statistically significant, does not represent a true clinically relevant change. To our knowledge, the MCID and MDC of the PRWE have not been determined in patients with distal radius fractures.

**Questions/Purposes:**

We asked: (1) What is the MCID of the PRWE score for patients with distal radius fractures? (2) What is the MDC of the PRWE?

**Methods:**

Our prospective cohort study included 102 patients with a distal radius fracture and a median age of 59 years (interquartile range [IQR], 48–66 years). All patients completed the PRWE questionnaire during each of two separate visits. At the second visit, patients were asked to indicate the degree of clinical change they appreciated since the previous visit. Accordingly, patients were categorized in two groups: (1) minimally improved or (2) no change. The groups were used to anchor the changes observed in the PRWE score to patients’ perspectives of what was clinically important. We determined the MCID using an anchor-based receiver operator characteristic method. In this context, the change in the PRWE score was considered a diagnostic test, and the anchor (minimally improved or no change as noted by the patients from visit to visit) was the gold standard. The optimal receiver operator characteristic cutoff point calculated with the Youden index reflected the value of the MCID.

**Results:**

In our study, the MCID of the PRWE was 11.5 points. The area under the curve was 0.54 (95% CI, 0.37–0.70) for the pain subscale and 0.71 (95% CI, 0.57−0.85) for the function subscale. We determined the MDC to be 11.0 points.

**Conclusions:**

We determined the MCID of the PRWE score for patients with distal radius fractures using the anchor-based approach and verified that the MDC of the PRWE was sufficiently small to detect our MCID.

**Clinical Relevance:**

We recommend using an improvement on the PRWE of more than 11.5 points as the smallest clinically relevant difference when evaluating the effects of treatments and when performing sample-size calculations on studies of distal radius fractures.

## Introduction

A frequently used outcome measure in distal radius fracture studies is the Patient-rated Wrist Evaluation (PRWE) score [8, 15]. The PRWE is a 15-item questionnaire designed to measure a patient’s wrist pain and disability. It consists of two subscales (pain and function) and has a score range from 0 (no disability) to 100 (severe disability). To recognize a treatment effect expressed as a change in PRWE score, it is important to be aware of the minimum clinically important difference (MCID) of the PRWE score. The MCID represents the smallest change in score that would be perceived by the patient as beneficial [4, 10, 21]. Consequently, a numeric change in score that is less than the MCID, even if statistically significant, does not represent a true clinically relevant change. Because the MCID defines a difference that is considered important to patients, the MCID also serves as the basis for estimating the necessary sample size in designing future studies [20].

Another important instrument is the minimum detectable change (MDC). The MDC is the smallest amount of change that falls outside the measurement error of an instrument. Therefore, any change smaller than the MDC could be the result of the variability of the questionnaire. To ensure that the MDC is sufficiently small to detect the MCID, the MCID should be greater than the MDC.

The MCID and the MDC of the PRWE have been examined in patients with chronic wrist conditions [12, 20, 22]; however, to our knowledge, they have not been determined in patients with a distal radius fracture.

Therefore, the purpose of our study was to determine the MCID and MDC of the PRWE score in patients with distal radius fractures.

## Patients and Methods

Our prospective cohort study was conducted alongside two ongoing clinical trials that are coordinated from our institution, an academic Level-1 trauma center in The Netherlands. The medical ethical review committee granted approval before initiation of this parallel study, without the need for informed consent from patient participants.

Patients for our cohort study were recruited from the two ongoing clinical trials between January 2011 and July 2014, during their first visit to the outpatient clinic. To increase the size of our cohort study population, we also recruited patients with distal radius fractures at the outpatient clinic who were not enrolled in the clinical trials. The patients who were not participants of the clinical trial were enrolled in our study between January 2014 and July 2014.

Our study population consisted of 102 patients with distal radius fractures. Patients were excluded if they: (1) did not want to complete the questionnaire at the outpatient clinic; (2) did not complete the anchor questions; (3) were unable to understand the study information; or (4) had sustained their distal radius fracture more than 1 year before their visit to the outpatient clinic.

Of the two concurrent clinical trials occurring during our prospective cohort study, the first trial [3] included 42 patients who underwent a study of two- and three-dimensional imaging. This trial provided 42 adult patients with intraarticular distal radius fractures who were treated with open reduction and internal fixation with a volar locking plate.

The second trial [25] randomized patients with displaced extraarticular distal radius fractures (AO types A2 and A3 [17]) between treatment with either open reduction and internal fixation with a volar locking plate or plaster immobilization. This trial provided 39 patients.

Additionally, during the first 6 months of 2014, we identified 55 patients who were not enrolled in either clinical trial but who were eligible for participation in our study. All adult patients with a distal radius fracture were eligible for inclusion, regardless of the type of treatment they received. After exclusion, an additional 21 patients with a distal radius fracture who were not enrolled in either of the two trials were included in our study cohort.

There are two methods to define the MCID: (1) a distribution-based and (2) an anchor-based approach [5]. The distribution-based approach is used to evaluate if the observed effect is attributable to true change or simply the variability of the questionnaire. It examines the distribution of observed scores in a group of patients. The magnitude of the effect is interpreted in relation to variation of the instrument [9]. In other words, is the observed effect attributable to true change or simply the variability of the questionnaire?

The anchor-based approach uses an external criterion (the anchor) to determine the MCID. Possible anchors include objective measurements, such as prehensile grip strength and ROM, or patient-reported anchor questions. The purpose of a patient-reported anchor question is to “anchor” the changes observed in the PRWE score to patients’ perspectives of what is clinically important [13].

Anchor-based methods to determine the MCID are preferred because an external criterion is used to define what is clinically important [7]; however, the anchor-based method does not take into account the measurement error of the instrument, so it is valuable to use the anchor- and distribution-based approaches [7]. To avoid confusion, the distribution-based method generally is referred to as minimum detectable change (MDC), and the anchor-based method as MCID [7]. We use the same terms to identify the methods.

Data were collected prospectively. Patients completed the Dutch version of the PRWE questionnaire during two visits at approximately 6 to 12 weeks and approximately 12 to 52 weeks after distal radius fracture injury.

**Fig. 1:**
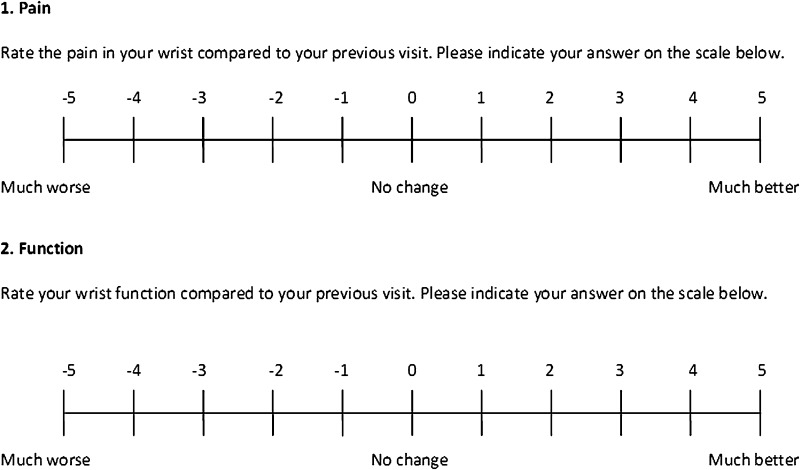
The global rating of change (GRC) scale used in the Patient-rated Wrist Evaluation (PRWE) questionnaire is shown. The anchor questions allowed patients to assess their current health status regarding wrist function and wrist pain, and compare their status with that of their previous visit.

At the second visit, patients were asked to indicate the degree of clinical change they had noticed since the previous visit for each domain (pain and function). Patients noted their answers on a global rating of change scale (GRC) from −5 (much worse) to +5 (much better) (Fig. 1) [11]. The purpose of this question was to “anchor” the changes observed in the PRWE score to patients’ perspectives regarding what is clinically important [13].

There is no consensus regarding the required sample size to determine the MCID [19]. We made a sample size estimation based on a conservatively estimated MCID of 12 points, with a SD of ± 14 [12, 20, 22]. To achieve an α of 0.05 and a power of 80%, we required 18 data points representing no change, and 18 data points representing minimal improvement.

### Statistical Methods

The number of questions not answered by patients comprised less than 5% for all items and were replaced with the mean score of the subscale according to the PRWE user manual [14]. PRWE scores were calculated for both subscales (pain and function) using the published algorithm [14]. The change in outcome was calculated as the difference between the last and the first scores. The change in score between visits was transformed such that improvement was indicated by a positive value. We reported medians and interquartile ranges (IQR) for nonparametric variables, and means (± SD) for normally distributed variables. The Kolmogorov-Smirnov test was used to determine if a variable was normally distributed. A p value of 0.05 or less was considered statistically significant. Data entry and analysis were performed using SPSS^®^ (Version 20.0; IBM Corp, Armonk, NY, USA) and RStudio Version 3.1.2; RStudio, Boston, MA, USA), with the package coefficient alpha.

### Determination of MDC

We calculated the MDC for the pain and function subscales separately and summed them to obtain the total MDC [7]. The MDCs were calculated as:

$$z\,{\text{score}}_{{\left( { 90\% } \right)}} \,*\, \, \surd 2 { }\,*\,{\text{Standard}}\;{\text{Error}}\;{\text{of}}\;{\text{Measurement}}_{\text{PRWE}}$$

A *z* score of 1.65 was chosen to reflect a 90% one-sided CI, similar to previous studies [12, 20]. The standard error of measurement is a measure of the instrument variability and takes into account the distribution of repeated measures on a questionnaire around the “true” score of a patient. For our study, the standard error of measurement was calculated by multiplying the SD (σ) of the PRWE score at the second followup, by the square root of 1, minus the reliability coefficient (*r*) of the instrument, or, in formula [1, 7]:

$${\text{Standard}}\;{\text{Error}}\;{\text{of}}\;{\text{Measurement}} = \sigma \,*\,\surd \, ( 1 { }{-}r)$$

The reliability coefficient is the overall consistency of an instrument. We used Cronbach’s alpha as a parameter of reliability [2]. Cronbach’s alpha is used to measure the internal consistency of a (sub)scale. Its value can range from 0 to 1.0, where greater than 0.7 indicates good internal consistency [2].

### Determination of the MCID

We calculated the MCID according the receiver operating characteristic (ROC) curve method [6, 12]. In this context, the change in PRWE score was considered a diagnostic test and the anchor was the gold standard [6]. The ROC curve plots the sensitivity against 1-specificity for all possible cutoff points of the change in PRWE score. The optimal ROC cutoff point is the value for which the sum of percentages of false positive and false negative classifications is smallest ([1-sensitivity] + [1-specificity]) [6]. This value represents the MCID. The area under the ROC curve reflects the ability of the change in PRWE score to differentiate between patients with and without clinically important change. The area under the ROC curve ranges from 0.5 to 1; a higher score indicates better discrimination.

Consistent with previous studies [22, 24], patients were categorized in five groups according to their answer to the anchor question: −5 to −4 (marked worsening); −3 to −2 (minimal worsening); −1 to 1 (no change); 2 to 3 (minimal improvement); and 4 to 5 (marked improvement). We calculated the MCID by plotting the ROC of the change in PRWE score for patients in the minimal-improvement group compared with patient scores in the no-change group.

**Fig. 2:**
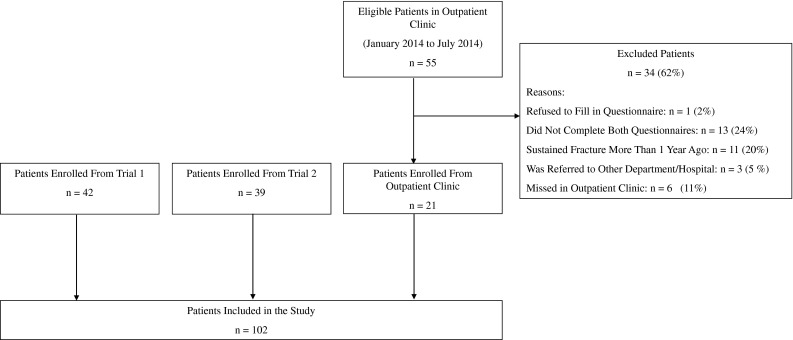
The flowchart shows patient selection methods used for the study.

We tested for significant score changes among patients who indicated they had experienced marked worsening, minimal worsening, no change, minimal improvement, and marked improvement, using the Kruskal-Wallis test. Nonsignificant differences among the five patient categories could suggest that the improvement categories were not sufficiently discriminative. The adequateness of the GRC scale was explored by quantifying the correlation between change in PRWE scores and the anchor questions using Spearman’s rho. Correlation coefficients were interpreted as negligible correlation (0–0.3); low correlation (0.3–0.5); moderate correlation (0.5–0.7); high correlation (0.7–0.9); or very high correlation (0.9–1.0) [16]. A total 102 patients were included in our study (Fig. 2). Patient characteristics are provided (Table 1).

**Table 1:** Characteristics of study population (n = 102)

Characteristic	Numbers
Age, median year (IQR)	59 (48–66)
Women, n (%)	71 (70)
Dominant hand affected, n (%)	50 (49)
AO fracture classification, n (%)	
A	56 (55)
B	11 (11)
C	35 (34)
Type of treatment, n (%)	
Open reduction and volar locking plate, n (%)	65 (64)
Plaster, n (%)	36 (35)
None*, n (%)	1 (1)
Weeks from trauma to first measurement, median (IQR)	8 (6–13)
Weeks between measurements, median (IQR)	8 (6–39)
Weeks from trauma to second measurement, median (IQR)	16 (13–52)
PRWE score at first measurement, median (IQR)	44 (21–63)
PRWE score at second measurement, median (IQR)	17 (4–45)

* Patient was treated elsewhere and the fracture was missed; IQR = interquartile range; PRWE = Patient-rated Wrist Evaluation.

## Results

### MCID of the PRWE for Patients with Distal Radius Fractures

The overall MCID was 11.5 points on the PRWE (Table 2). For the pain subscale, 20% of the patients (20/102) indicated they had experienced minimal improvement and 37% (38/102) experienced no change. The area under the ROC curve of the change in PRWE score to differentiate between patients with minimal improvement in pain and patients with no change in pain was 0.54 (95% CI, 0.37–0.70). For the function subscale, 24% of the patients (24/102) reported minimal improvement in function and 34% (35/102) experienced no change. The area under the ROC curve of the change in PRWE score to differentiate between patients with minimal improvement in function and no change in function was 0.71 (95% CI, 0.57−0.85).

**Table 2:** MCID and the MDC of the PRWE score

Subscale	MCID*	MDC*
PRWE pain	1.5	6.5
PRWE function	10	4.5
PRWE total	11.5	11.0

* Units are expressed in points on the PRWE score; MCID = minimal clinically important difference; MDC = minimal detectable change; PRWE = Patient-rated Wrist Evaluation.

### MDC of the PRWE

The MDC was 11.0 points. The majority of patients reported marked improvement (Table 3) and the PRWE scores between the first and the second measurements differed (p < 0.001; Wilcoxon signed rank test). For the pain subscale, 40 patients reported marked improvement (change in PRWE, 9.5; IQR, 5.0–16.0), and 20 patients had minimal improvement (change in PRWE, 5.0; IQR, −1.8 to 10.7). For the function subscale, 41 patients reported marked improvement (change in PRWE, 12.5; IQR, 5.8–19.7), and 24 patients had minimal improvement (change in PRWE, 10.8; IQR, 3.6–18.8).

**Table 3:** Changes in Patient-rated Wrist Evaluation scores

	Anchor category					
	Marked worsening	Minimal worsening	No change	Minimal improvement	Marked improvement	p value*
	(n = 1)	(n = 3)	(n = 38)	(n = 20)	(n = 40)	
Pain subscale						
Change, median (IQR)	Not applicable	−8.0 (−15 to 5.0)	4.0 (0.0–10.9)	5.0 (−1.8 to 10.7)	9.5 (5.0–16.0)	0.001

	Anchor category					
	Marked worsening	Minimal worsening	No change	Minimal improvement	Marked improvement	p value*
	(n = 1)	(n = 1)	(n = 35)	(n = 24)	(n = 41)	
Function subscale						
Change, median (IQR)	Not applicable	Not applicable	3.5 (0.0–6.5)	10.8 (3.6–18.8)	12.5 (5.8–19.7)	<0.001

* Kruskal-Wall test used for determining significance; IQR, interquartile range.

There were significant differences in the changes in PRWE scores among patients who indicated they had experienced marked worsening, minimal worsening, no change, minimal improvement, or marked improvement in pain (p = 0.001, Kruskal-Wallis test), suggesting sufficiently discriminative categories (Table 3). There also were significant differences in the changes in PRWE scores among the categories of the function subscale (p < 0.001, Kruskal-Wallis test).

There was correlation between the change in PRWE scores for the pain subscale and the GRC categories, confirming the adequacy of the GRC (correlation coefficient = 0.39; two-tailed p < 0.001). The correlation between the change in PRWE score and GRC categories for function was similar (correlation coefficient = 0.34; two-tailed p = 0.001). Reliability coefficients (Cronbach’s alpha) were 0.98 for the pain subscale and 0.95 for the function subscale, indicating good internal consistency of the questionnaire.

## Discussion

The PRWE score is a well-accepted measure of patient functional outcome after distal radius fracture [8]. Knowledge of the MCID of the PRWE provides a useful benchmark to interpret study results and a basis for sample-size calculations. Three previous studies have examined the MCID of the PRWE; however, to our knowledge, no such study has examined patients with distal radius fractures [12, 20, 22]. Some authors advocate that the MCID is not a universal fixed attribute and cannot be applied across patient populations or disease-specific states [4, 19, 26, 27]. The MCID can fluctuate based on what is interpreted as important to the patient; therefore, patients with chronic wrist conditions may have other expectations from treatment than patients with an acute condition. Patients who sustain a distal radius fracture generally have their healthy wrist become immobilized, are in pain, and experience a (temporary) complete loss of wrist function. Their standard of comparison is likely not the painful situation at the beginning of treatment for the fracture, but their status before the injury [18]. In general, these patients expect complete recovery, which could entail that they require different changes in PRWE scores to appreciate clinical improvement.

In our patients, the MCID was 11.5 points, which was just outside the measurement error (MDC) of the PRWE score.

Our study had several limitations. The majority of patients were selected from one of two ongoing clinical trials coordinated from our institution. Owing to the nature of the trials, the patients in our study were part of a more selective group of patients. All patients had a sustained displaced distal radius fracture and consented to participate in a randomized controlled trial. Such a select group of patients may limit the generalizability of our results. Other limitations pertain to the various approaches for determining the MCID. For example, a limitation of the anchor-based approach is that it does not take measurement precision into account [5, 7]; therefore, the MCID determined potentially can be within the measurement error of the questionnaire. By determining the MDC, it becomes possible to judge whether the MDC of a measurement instrument is sufficiently small to detect the MCID [7]. In our study, the MDC was 11 points, therefore the MCID we determined was outside the measurement error of the questionnaire. Another limitation of the anchor-based method is the possibility of recall bias [23] Recall bias implies that patients are unable to recall their initial state at the time of injury. Recall bias was present in our study, illustrated by the low correlation we found between the change in scores and the anchor questions; however, none of the patients gave contradicting answers (indicating worsening status in response to the anchor questions while their PRWE score had improved, or vice versa). The relatively short duration between measurements (8 weeks) might have contributed to this. An increased duration of followup in a study is associated with larger estimates of the MCID [24], therefore we chose to limit the followup to 1 year, similar to that in a previous study on the MCID of the PRWE in patients with traumatic upper-extremity conditions [22].

The MCID for our patients was 11.5 points, which was lower than previously determined MCIDs. Three previous studies have examined the MCID of the PRWE. Schmitt and Di Fabio [20] reported a MCID of 24 points in a cohort of 211 patients, however their patients predominantly had shoulder pain, and the PRWE is not intended for patients with shoulder injuries. The second study, by Sorensen et al. [22], included 102 patients with a traumatic upper-extremity conditions such as isolated tendinitis, arthritis, and nerve compression syndrome. The MCID in that study was 14 points. The third study included 31 patients who underwent ulnar-shortening osteotomy for ulnar impaction syndrome and the MCID was 17 points [12].

The MDC is the smallest change in score that likely reflects true change rather than measurement error. It shows which changes fall outside the measurement error of the health status measurement (based on, for instance, internal validity or test-retest reliability) [7]. To ensure that the MDC is sufficiently small to detect the MCID, it should be greater than the MDC. We found an MDC of 11.0 points, similar to the MDCs reported by Kim and Park (7.7 points) [12] and Schmitt and Di Fabio (12.2 points) [20]. This value for the MDC indicates that the PRWE questionnaire is able to detect changes as small 11.0 points, therefore the PRWE should be able to detect the MCID we determined.

In our prospective cohort study, we determined the MCID of the PRWE for patients with distal radius fractures using the anchor-based approach and verified that the MDC of the PRWE was sufficiently small to detect our MCID. The MCID is not a value that can be used to classify individual treatment results, but rather a method to put group-level treatment effects in perspective. We recommend using an improvement on the PRWE of more than 11.5 points as the smallest clinically relevant difference when evaluating the effects of treatments and when performing sample-size calculations on studies of distal radius fractures.

## References

[1] Beaton DE, Boers M, Wells GA (2002). Many faces of the minimal clinically important difference (MCID): a literature review and directions for future research. Curr Opin Rheumatol..

[2] Beaton DE, Bombardier C, Katz JN, Wright JG, Wells G, Boers M, Strand V, Shea B (2001). Looking for important change/differences in studies of responsiveness. OMERACT MCID Working Group. Outcome measures in rheumatology: minimal clinically important difference. J Rheumatol..

[3] Beerekamp MS, Ubbink DT, Maas M, Luitse JS, Kloen P, Blokhuis TJ, Segers MJ, Marmor M, Schep NW, Dijkgraaf MG, Goslings JC (2011). Project group of the EF3X-trial. Fracture surgery of the extremities with the intra-operative use of 3D-RX: q randomized multicenter trial (EF3X-trial). BMC Musculoskelet Disord..

[4] Calfee RP, Adams AA (2012). Clinical research and patient-rated outcome measures in hand surgery. J Hand Surg Am..

[5] Crosby RD, Kolotkin RL, Williams GR (2003). Defining clinically meaningful change in health-related quality of life. J Clin Epidemiol..

[6] de Vet HC, Ostelo RW, Terwee CB, van der Roer N, Knol DL, Beckerman H, Boers M, Bouter LM (2007). Minimally important change determined by a visual method integrating an anchor-based and a distribution-based approach. Qual Life Res..

[7] de Vet HC, Terwee CB, Ostelo RW, Beckerman H, Knol DL, Bouter LM (2006). Minimal changes in health status questionnaires: distinction between minimally detectable change and minimally important change. Health Qual Life Outcomes..

[8] Gupta S, Halai M, Al-Maiyah M, Muller S (2014). Which measure should be used to assess the patient’s functional outcome after distal radius fracture?. Acta Orthop Belg..

[9] Guyatt GH, Osoba D, Wu AW, Wyrwich KW, Norman GR, Clinical Significance Consensus Meeting Group (2002). Methods to explain the clinical significance of health status measures. Mayo Clin Proc..

[10] Jaeschke R, Singer J, Guyatt GH (1989). Measurement of health status: ascertaining the minimal clinically important difference. Control Clin Trials..

[11] Kamper SJ, Maher CG, Mackay G (2009). Global rating of change scales: a review of strengths and weaknesses and considerations for design. J Man Manip Ther..

[12] Kim JK, Park ES (2013). Comparative responsiveness and minimal clinically important differences for idiopathic ulnar impaction syndrome. Clin Orthop Relat Res..

[13] Lydick E, Epstein RS (1993). Interpretation of quality of life changes. Qual Life Res..

[14] MacDermid JC. The patient-rated wrist evaluation (PRWE) user manual.2007. Available at: http://srs-mcmaster.ca/wp-content/uploads/2015/05/English-PRWE-User-Manual.pdf. Accessed September 18, 2014.

[15] MacDermid JC, Turgeon T, Richards RS, Beadle M, Roth JH (1998). Patient rating of wrist pain and disability: a reliable and valid measurement tool. J Orthop Trauma..

[16] Mukaka MM (2012). Statistics corner: a guide to appropriate use of correlation coefficient in medical research. Malawi Med J..

[17] Müller ME, Nazarian S, Koch P, Schatzker J (1990). The Comprehensive Classification of Fractures of Long Bones.

[18] Norman GR, Sloan JA, Wyrwich KW (2003). Interpretation of changes in health-related quality of life the remarkable universality of half a standard deviation. Med Care..

[19] Revicki D, Hays RD, Cella D, Sloan J (2008). Recommended methods for determining responsiveness and minimally important differences for patient-reported outcomes. J Clin Epidemiol..

[20] Schmitt JS, Di Fabio RP (2004). Reliable change and minimum important difference (MID) proportions facilitated group responsiveness comparisons using individual threshold criteria. J Clin Epidemiol..

[21] Smith MV, Calfee RP, Baumgarten KM, Brophy RH, Wright RW (2012). Upper extremity-specific measures of disability and outcomes in orthopaedic surgery. J Bone Joint Surg Am..

[22] Sorensen AA, Howard D, Tan WH, Ketchersid J, Calfee RP (2013). Minimal clinically important differences of 3 patient-rated outcomes instruments. J Hand Surg Am..

[23] Stratford PW, Binkley JM, Riddle DL, Guyatt GH (1998). Sensitivity to change of the Roland-Morris back pain questionnaire: part 1. Phys Ther..

[24] Tashjian RZ, Deloach J, Green A, Porucznik CA, Powell AP (2010). Minimal clinically important differences in ASES and simple shoulder test scores after nonoperative treatment of rotator cuff disease. J Bone Joint Surg Am..

[25] Walenkamp MM, Goslings JC, Beumer A, Haverlag R, Leenhouts PA, Verleisdonk EJ, Liem RS, Sintenie JB, Bronkhorst MW, Winkelhagen J, Schep NW (2014). Surgery versus conservative treatment in patients with type A distal radius fractures: a randomized controlled trial. BMC Musculoskelet Disord..

[26] Wang YC, Hart DL, Stratford PW, Mioduski JE (2011). Baseline dependency of minimal clinically important improvement. Phys Ther..

[27] Wright JG (1996). The minimal important difference: who’s to say what is important?. J Clin Epidemiol..

